# IL-17A-Mediated Excessive Autophagy Aggravated Neuronal Ischemic Injuries via Src-PP2B-mTOR Pathway

**DOI:** 10.3389/fimmu.2019.02952

**Published:** 2019-12-20

**Authors:** Ting Liu, Song Han, Qingqing Dai, Jiayin Zheng, Cui Liu, Shujuan Li, Junfa Li

**Affiliations:** ^1^Department of Neurobiology and Center of Stroke, Beijing Institute for Brain Disorders, School of Basic Medical Science, Capital Medical University, Beijing, China; ^2^Department of Neurology, Beijing Chao-Yang Hospital, Capital Medical University, Beijing, China

**Keywords:** ischemic stroke, IL-17A, autophagy, OGD/R, Src-PP2B-mTOR pathway

## Abstract

We previously reported that astrocyte-derived proinflammatory cytokine interleukin (IL)-17A could aggravate neuronal ischemic injuries and strength autophagy both in oxygen-glucose deprivation (OGD)/reoxygenation (R)-treated neurons and peri-infarct region of mice with middle cerebral artery occlusion (MCAO)/reperfusion (R)-simulated ischemic stroke. In this study, the role and molecular mechanism of IL-17A in autophagy were further explored under ischemic condition. We found that exogenous addition of rmIL-17A remarkably (*P* < 0.001) decreased cell viability, which companying with the increases of LC3 II accumulation (*P* < 0.05 or 0.01) and Beclin 1 levels (*P* < 0.05 or 0.001), and reduction of p62 levels (*P* < 0.01 or 0.001) in OGD/R-treated cortical neurons (*n* = 6). The levels of P-mTOR (Ser 2448) (*P* < 0.001) and P-S6 (Ser 240/244) (*P* < 0.01) significantly decreased without the involvement of Akt, ERK1/2 and AMPK in cortical neurons under rmIL-17A and OGD/R treatments (*n* = 6). Interestingly, the co-IP analysis exhibited that PP2B and mTOR could be reciprocally immunoprecipitated; and the addition of rmIL-17A increased their interactions, PP2B activities (*P* < 0.001), P-Src (*P* < 0.001), and P-PLCγ1 (*P* < 0.01) levels in OGD/R-treated neurons (*n* = 6 or 5). The PP2B inhibitor Cyclosporin A blocked the induction of excessive autophagy (*P* < 0.05 or <0.001) and increased cell viability (*P* < 0.001) after OGD/R and rmIL-17A treatments (*n* = 6). In addition, the ICV injection of IL-17A neutralizing mAb could attenuate autophagy levels (*P* < 0.01 or 0.001, *n* = 6) and improve neurological functions (*P* < 0.01 or 0.001, *n* = 10) of mice after 1 h MCAO/R 24 h or 7 d. These results suggested that IL-17A-mediated excessive autophagy aggravates neuronal ischemic injuries via Src-PP2B-mTOR pathway, and IL-17A neutralization may provide a potential therapeutic effect for ischemic stroke.

## Introduction

As the leading cause of death, ischemic stroke (>80% of stroke) is a medical emergency with high morbidity and mortality (1, 2). It causes multiple pathophysiological events including mitochondrial response, excitotoxicity, protein misfolding and immune response, which lead to the delayed neuronal loss (3). These evidences providea range of molecular mechanisms that are potential targets for intervention. Interleukin (IL)-17A, a potent proinflammatory cytokine, is believed to have a specific role in the delayed phase of the post-infarct inflammatory response (4). We have reported that the astrocyte-derived IL-17A reached the peak at 12 h or 3 d reperfusion (R) in peri-infarct region/cerebrospinal fluid (CSF) and serum of mice after 1 h middle cerebral artery occlusion (MCAO), respectively (5). In addition, the injection of IL-17A neutralizing monoclonal antibody (mAb) could reduce the infarct volume and improve neurological outcome of mice with ischemic stroke (6, 7). However, how this intervention functions still remain unclear.

Autophagy is a self-protecting cellular process, through which misfolded proteins, protein aggregates, and dysfunctional organelles are degraded into metabolic constituents and recycled for maintaining cellular homeostasis. Current reports indicate that autophagy is controlled by a complicated signaling network (8). The mechanistic target of rapamycin (mTOR) is involved in the negative regulation of autophagy, and its major upstream regulators are Akt, extracellular signal-regulated kinase (ERK) and adenosine 5'-monophosphate-activated protein kinase (AMPK) (9). Autophagy is closely related with inflammatory responses and the relationship between them is extraordinarily complicated (10). Following engagement of the IL-17RA/C receptor complex in neurons by IL-17A, non-receptor tyrosine kinases could be recruited to the SEFIR domain in the receptor complex, resulting in the consecutive recruitment of downstream signaling pathways. IL-17A could induce autophagy via Janus kinase/signal transducer (JAK2/STAT3) and c-Jun N-terminal kinase (JNK) signaling pathway in human SMMC-7721 cells and osteoclast precursors (OCPs), respectively (11, 12). On the contrary, IL-17A inhibited autophagy via TAB2/TAB3-p38 mitogen-activated protein kinase pathways and mTOR signaling in Hepatocellular carcinoma (HCC) cells and keratinocytes (13, 14).

The activation of autophagy was witnessed in neurons after oxygen-glucose deprivation (OGD)/reoxygenation (R) and MCAO/R treatments (15–17). However, the role of autophagy and IL-17A in ischemic stroke are still ambiguous. In this study, we explored the exact role and molecular mechanism of IL-17A in neuronal autophagy after ischemic stroke both *in vivo* and *in vitro*.

## Materials and Methods

All animal procedures were performed strictly in accordance with the recommendations in the guide for the care and use of Laboratory Animals of the National Institutes of Health and approved by the Experimental Animal Ethics Committee of the Capital Medical University (SCXK2016-0006).

### MCAO-Induced Ischemic Stroke Mouse Model

Adult male C57 BL/6 J mice (6–8 W, 18–22 g) were purchased from the Jackson Laboratory (Bar. Harbor, ME, USA) and maintained in the Experimental Animal Center of Capital Medical University, PR China. They were housed under constant temperature (23 ± 2°C), humidity (40–70%) and maintained on a 12-h light/dark cycle with food and water available.

Sixty-Four C57 BL/6 J mice were randomly divided into four groups as follows: Sham (*n* = 16), MCAO (*n* = 16), IgG isotype (*n* = 16) and MCAO + IL-17A neutralizing monoclonal antibody (mAb, *n* = 16). After 1 h MCAO/R 24 h, the mice from Sham (*n* = 6), MCAO (*n* = 6), IgG isotype (*n* = 6), and MCAO + IL-17A mAb (*n* = 6) groups were used to examine the expressions of autophagy-related proteins by using immunoblotting. The left mice from Sham (*n* = 10), MCAO (*n* = 10), IgG isotype (*n* = 10), and MCAO + IL-17A mAb (*n* = 10) groups after 7 days' reperfusions were designed to evaluate the neurological outcome.

The MCAO/R-induced ischemic stroke mouse model was prepared as previously described (7, 18, 19). Mice were anesthetized with sodium pentobarbital (60 mg/kg) intraperitoneally (i.p.), and the body temperature was maintained at 36.5–37.5°C by using a heating pad during the surgery. Then the left common carotid artery (CCA), the left external carotid artery (ECA), and the internal carotid artery (ICA) were surgically exposed via a ventral midline incision. Next, the CCA and ECA were ligated, and the ICA was clipped by using microvascular aneurysm clips. After an arteriotomy was made in the ECA, a soft silicone coated surgical nylon monofilament suture (0.23 mm in diameter; 3.0 cm in length, RWD Life Science, China) was gently inserted into the ICA through the ECA to occlude the middle cerebral artery (MCA, a point approximately 12.0 mm distal to the carotid bifurcation). After 1 h occlusion, the suture was carefully withdrawn to restore blood supply and the ECA was permanently ligated to prevent the incision from bleeding. Finally, reperfusion was achieved by loosening the temporary ligation on the CCA. Post-operative mice were placed in a temperature controlled cage with regular observation for 24 h. Laser Doppler flowmetry (Perimed PeriFlux system 5000, Jarfalla, Stockholm, Sweden) was employed to monitor cerebral blood flow (CBF) during MCAO surgery and IL-17A mAb injection and to ensure that the blood circulation was occluded completely. Regional CBF decreased by 80% in mice after MCAO and restored totally after the suture was removed 1 h later. In the Sham group, mice received the same procedure, without inserting the nylon monofilament to occlude the MCA.

### Intracerebroventricular Injection of IL-17A Neutralizing mAb

The IL-17A neutralizing mAb (2.0 μg, #560268; Becton Dickinson, New Jersey, USA) or mouse IgG isotype (2.0 μg) was injected into the intracerebroventricle (ICV) of mice at 3 h after MCAO. The ICV injection was performed as previously described (20). Briefly, the anesthetized mice (sodium pentobarbital, 70 mg/kg, i.p.) were placed upon a stereotaxic frame. The cannula (28-G, inner diameter 0.18 mm; outer diameter 0.36 mm) was lowered into the right cerebral ventricle according to the following coordinates: 0.5 mm posterior and 1.0 mm lateral to bregma, and 3.2 mm below the skull surface. The total volume of IL-17A neutralizing mAb and IgG isotype are 2 μl. The injection was operated at the rate of 0.2 μl/min for 10 mins, then retained the needle for another 10 mins.

### Evaluation of Neurological Functions

The neurological functions of MCAO mice after 7 d reperfusions were evaluated by an observer who was blinded to the experiment design. Neurobehavioral scores were measured according to the neurological disability status scale (NDSS) reported by Rodriguez et al. (21), which has 10 progressive steps from 0 (normal) to 10 (death). The detailed criteria were as follows: 0, no neurological dysfunction; 2, slight dysfunction in mobility and presence of passivity; 4, moderate neurological dysfunction; 6, more handicapped animals with more marked hypomobility, circling, tremor, jerks and/or convulsions, forelimb flexion and moderate motor incoordination; 8, respiratory distress and total incapacity to move/coordinate; and 10 represents death due to 1 h MCAO/R 7 d. In all cases, where criteria for the precise score were not met, the nearest appropriate number was recorded: 1, 3, 5, 7, and 9.

Neurological deficits were evaluated on a modified scoring system suggested by Ding et al. (22) as follows: 0, no neurological deficit; (1) Difficulty in fully extending the contralateral forelimb; (2) Failure to extend contralateral forelimb; (3) Mild circling to the contralateral side; (4) Severe circling; and (5) Falling to the contralateral side.

Corner test was examined as described by Li et al. (23). The mouse was placed into a corner of 30° that was formed by moving two cardboard pieces in front of its nose. When both sides of the vibrissae were stimulated by the two boards, the mouse reared forward and upward, then turned back to face the open end. Twenty trials were performed and the laterality index was calculated using the formula (number of left turns -number of right turns)/10. Only turns involving full rearing along either board were counted.

Beam balance test was employed to evaluate motor coordination and balance. Mice were trained to traverse a horizontal beam 0.7 cm wide, 120 cm long and 40 cm above the floor within 15 s, and the test was performed at 1 h MCAO/R 7d. Those mice fail to pass through the rod within 15 s were eliminated from the experiment after 3 days of training. The score criteria were as follows: 0, mice can't stay on the beam; 1, mice can stay on the beam, but can't move; 2, mice tried to pass through the beam but failed and dropped midway; 3, mice passed through the beam with more than 50% foot slips; 4, mice traverse the beam successfully with fewer than 50% foot slips; 5, mice passed the beam successfully with only one foot slip; 6, the rat traverse the beam without foot slips.

To evaluate motor coordination and balance, rotarod test from 4 to 40 rpm over a time course of 5 min was operated at 1 h MCAO/R 7d. In brief, mice were placed on an automated accelerating rotating rod (LE8200, Panlab Harvard Apparatus, USA) and their latency to fall off the rod was recorded. Preoperative training was carried out for 3 days with 3 daily trials; only those mice able to remain on the rod for 5 min at 40 rpm were subjected to MCAO surgery. Postoperative testing was performed at 7 days after MCAO, the mice was given 3 trials at 40 rpm per day and the average time (in seconds) spent on the rod was calculated for analysis.

### Primary Cortical Neuron Culture

The newborn 24 h C57 BL/6 J mice were rapidly decapitated and the cerebral cortices were separated. After removing the meninges and blood vessels, the cortices were cut into pieces of 0.2 cm^3^. Cortical neurons were dissociated with 0.25% trypsin-EDTA (25200-056, Gibco, Grand Island, USA) and seeded onto six-well plates at a density of 1 × 10^6^ cells/well. Cortical neurons were cultured in Dulbecco's Modified Eagle Medium (G11995500BT, Gibco, Beijing, China), which contained 10% horse serum (16050-122, Gibco, Grand Island, NY, USA), 10% fetal bovine serum (10099-141, Gibco, Grand Island, USA), 1% penicillin-streptomycin solution (15070063, Life Technologies, Carlsbad, ON, Canada), and 0.25% L-glutamine (25030-081, Gibco, Grand Island, USA). The medium was replaced by Neurobasal Medium (21103-040, Gibco, Grand Island, USA) containing 2% B27 supplement (17504-044, Gibco, Grand Island, USA) after 4 h. Half of the medium was changed every 72 h.

### OGD/R Model

The 1 h OGD/24 h R model was employed to simulate ischemia/reperfusion injury *in vitro*. When the neurons were cultured for 7 days, the medium was changed to glucose-free DMEM (11966-025, Gibco, Grand Island, USA) and placed in a 37°C hypoxia incubator (Thermo Scientific, Marietta, OH, USA) under hypoxic conditions (2% O_2_/5% CO_2_/93% N_2_) for 1 h. After that, glucose-free DMEM was replaced by Neurobasal Medium, which contained 2% B27 supplement under normoxic condition 5% CO_2_/21% O_2_/74% N_2_ for 24 h reoxygenation. Recombinant mouse (rm) IL-17A (250 ng/mL, 421-ML-025/CF, R&D System, MN), 3-MA (5 mM, S2767, Selleck Chemicals), Bafilomycin A1 (BafA1) (100 nM, S1413, Selleck Chemicals) and CsA (100 nM, S2286, Selleck Chemicals) dissolved in 4 mM HCL, H_2_O or DMSO, respectively, were added into the medium during OGD/R treatment. rmIL-17A dissolved in 40 μM HCL was used as vehicle group.

### Cell Viability Assay

To estimate the effect of rmIL-17A and autophagy inhibitors (3-MA and Baf A1) on the survival rate of primary cortical neurons after OGD/R treatment, the cells were planted at a density of 1 × 10^4^ cells/well on a 96-well plate. The cell culture and OGD model were same as above mentioned. After 1 h OGD/24 h R, Cell Titer 96 Aqueous One Solution Cell Proliferation Assay (G3580, Promega, Madison, WI, USA) was employed according to the manufacturer's instructions to detect the overall survival rate of the cells.

### PP2B Activity

PP2B Cellular Activity Assay Kit (BML-AK816, Enzo Life Sciences, Farmingdale, NY, USA) was used to measure cellular PP2B phosphatase activity. After 1 h OGD/24 h R, cells planted on six-well plates were washed in ice-cold TBS (20 mM Tris, pH 7.2, 150 mM NaCl). Cells were dissociated in 500 ul Lysis buffer with protease inhibitors and centrifuged at 100,000 g for 45 min at 4°C. To remove free phosphate, the high-speed supernatant extracts were desalted by gel filtration. The extract samples were estimated using PP2B phosphatase assay kit (BML-AK804, Enzo Life Sciences) as described in the instruction manual. PP2B activity was assessed by measuring absorbance at 620 nm and normalized using the controls.

### Immunoprecipitation Assays

Cells were lysed in IP buffer A (50 mM Tris–HCl (pH 7.5) containing 2 mM EDTA, 2 mM EGTA, 5 μg/μL each of leupeptin, aprotinin, pepstatin A and chymostatin, 50 nM okadaic acid, 5 mM sodium pyrophosphate, 100 μM sodium vanadate, 1 mM DTT, 50 mM KF, 5 M iodoacetamide) and then incubated with 4 μg antibody against mTOR/PP2B or IgG in a spin column from Protein G Immunoprecipitation Kit (IP50, Sigma-Aldrich, St. Louis, MO, USA) at 4°C overnight with constant rotation. After incubation, 30 μl washed Protein G Agarose were transferred to the lysate and incubated at 4°C overnight with constant rotation. The immunoprecipitates was resolved in buffer [100 mM Glycine, 1.5 M Tris-HCl (pH 8.0), 1 × loading buffer] and centrifuged. The flow-through was collected and heated at 95°C for 5 min and proceed for immunoblotting.

### Immunofluorescent Staining

After OGD treatment, neurons were fixed in 4% paraformaldehyde for 30 min at 25°C, washed four times with PBS, and then blocked with buffer (8% goat serum + 0.2% Triton X100 (101875278, Sigma-Aldrich, St. Louis, MO, USA) in PBS) for 1 h at 37°C. Neurons were incubated with mouse anti-LC3 (SAB1305552, Sigma-Aldrich) at 4°C overnight. After washing, the secondary Alexa Fluor 488-conjugated goat anti-mouse IgG (Invitrogen, Carlsbad, CA, USA) was added and incubated for 2 h at 37°C. Finally, cells were mounted with ProLong Gold antifade Mountant with DAPI (P36934, Life Technologies, Carlsbad, ON, Canada) and imaged using a Leica SP8 microscope with 63 × 1.4 Numerical Aperture (NA) oil objective lens (Leica, Wetzlar, Germany).

### Immunoblotting

After 1 h OGD/24 h R, Cells in six-well plates were rinsed three times with PBS and homogenized in Buffer C (50 mM Tris–HCl (pH 7.5) containing 2 mM EDTA, 2 mM EGTA, 5 μg/μL each of leupeptin, aprotinin, pepstatin A and chymostatin, 50 nM okadaic acid, 5 mM sodium pyrophosphate, 100 μM sodium vanadate, 1 mM DTT, 50 mM KF, 2% sodium dodecyl sulfate) and sonicated. Protein concentration was quantified via BCA protein assay kit (23225, Pierce Company, Rockford, IL 61101, USA), using albumin diluted in Buffer C as standard.

Samples loaded with equal amount of proteins (30 μg) were electrophoresed on 8–10% sodium dodecyl sulfate polyacrylamide gel (SDS-PAGE) and transferred onto polyvinylidene difluoride (PVDF) membrane (10600021, GE Healthcare, Buckinghamshire, UK). Blocking was performed in 10% non-fat milk in Tween/Tris-buffered salt solution(TTBS, 20 mM Tris-Cl, pH 7.5, 0.15 M NaCl, and 0.05% Tween-20) for 1 h and membranes were incubated with primary antibodies overnight at 4°C. Membranes were incubated in Horseradish peroxidase-conjugated goat anti-mouse or anti-rabbit IgG (1:4,000, Thermo Scientific, Marietta, OH, USA) for 1 h. Chemiluminescencent HRP substrate (90719, Millipore, Billerica, MA 01821, USA) was employed to detect the signals and proteins were visualized using Fusion-Capt Advance software on FUSION FX (Vilber Lourmat, Collégien, France). The primary antibodies used were LC3A/B (12741, Cell Signaling Technology, Danvers, MA, USA), Beclin 1(1:1,000,11306-1-AP, Proteintech, Rosemont, IL, USA), p62 (1:1,000, 5114, Cell Signaling Technology), P-mTOR (Ser 2448, 1:1,000, 5536, Cell Signaling Technology), mammalian target of rapamycin (mTOR) (1:1,000, 2983, Cell Signaling Technology), P-S6 (Ser 240/244, 1:1,000, 2215, Cell Signaling Technology), S6 Ribosomal Protein (1:1,000, 2317, Cell Signaling Technology), P-Akt (Thr 308,1:1,000, 9275, Cell Signaling Technology), Akt (1:1,000, 4691, Cell Signaling Technology), P-ERK (Tyr 202/Tyr 204, 1:1,000, 9101, Cell Signaling Technology), ERK (1:1,000, 9102, Cell Signaling Technology), P-AMPK (Thr 172, 1:1,000, 2535, Cell Signaling Technology), AMPK (1:1,000, 07-350, Millipore, St. Louis, MA, USA), P-Src Family (Tyr 416, 1:1,000, 6943, Cell Signaling Technology), Src (1:1,000, 2109, Cell Signaling Technology), P-PLCγ1 (Tyr 783, 1:1,000, 2821, Cell Signaling Technology), PLCγ1 (1:1,000, 2822, Cell Signaling Technology), β-actin (1:10,000, 60008-1-Ig, Proteintech, Rosemont, IL, USA), β-tubulin (1:20,000, 66240-1-Ig, Proteintech, Rosemont, IL, USA).

### Statistical Analysis

Data were represented as mean ± SEM. Immunoblots were quantified using Image J. GraphPad Prism version 7.0 (GraphPad Software, La Jolla, CA, USA) was used for data analysis. Statistical analysis was performed using one-way or two-way analysis of variance (ANOVA) followed by all pairwise multiple comparison procedures using Bonferroni test. *P* < 0.05 was considered statistically significant. For the *in vitro* experiments, the N number represents 6 times of primary neuronal preparation under the same culture conditions; while for the *in vivo* experiments, the N number represents 6 mice under the same conditions.

## Results

### rmIL-17A Aggravates OGD/R-Induced Neuronal Ischemic Injuries Through Enhancing Autophagy Levels

**Figure 1:**
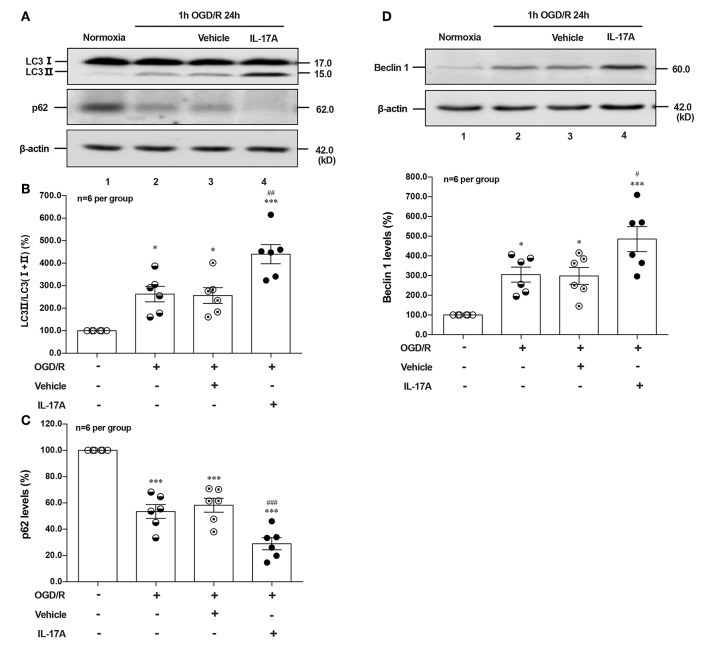
Effect of rmIL-17A on autophagy levels in primary cortical neurons after OGD/R treatment. The representative image and quantitative analysis results of Western blot analysis showed that rmIL-17A could significantly increase the ratio of LC3 II/LC3 (I+II) and Beclin 1 protein levels in 1 h OGD/R 24 h-treated primary cortical neurons **(A,B,D)** compared with normoxia groups. In addition, rmIL-17A could strengthen the degradation of p62 in cortical neurons **(A,C)** after 1 h OGD/R 24 h treatment (*n* = 6 per group). *P < 0.05, ***P < 0.001 vs. corresponding Normoxia group; ^#^*P* < 0.05, ^##^*P* < 0.01, ^###^*P* < 0.001 vs. corresponding vehicle group.

To determine the effect of IL-17A on autophagy after OGD/R treatment, the LC3 conversion (LC3 I to LC3 II), p62 and Beclin 1 protein levels were observed in 1 h OGD/R 24 h-treated neurons. As shown in Figure 1, rmIL-17A could significantly increase the conversion ratio of LC3 II/total LC3 and Beclin 1 protein levels in 1 h OGD/R 24 h-treated primary cultured cortical neurons (A, B, and D) when compared with that of normoxic groups. Similarly, rmIL-17A could strengthen the degradation of p62 in cortical neurons (A and C) after 1 h OGD/R 24 h treatment. In line with this, IL-17A increased the number of LC3 puncta in neurons after OGD treatment (**Figure 5H**). These results suggested that IL-17A could enhance the autophagy levels in ischemic neurons.

**Figure 2:**
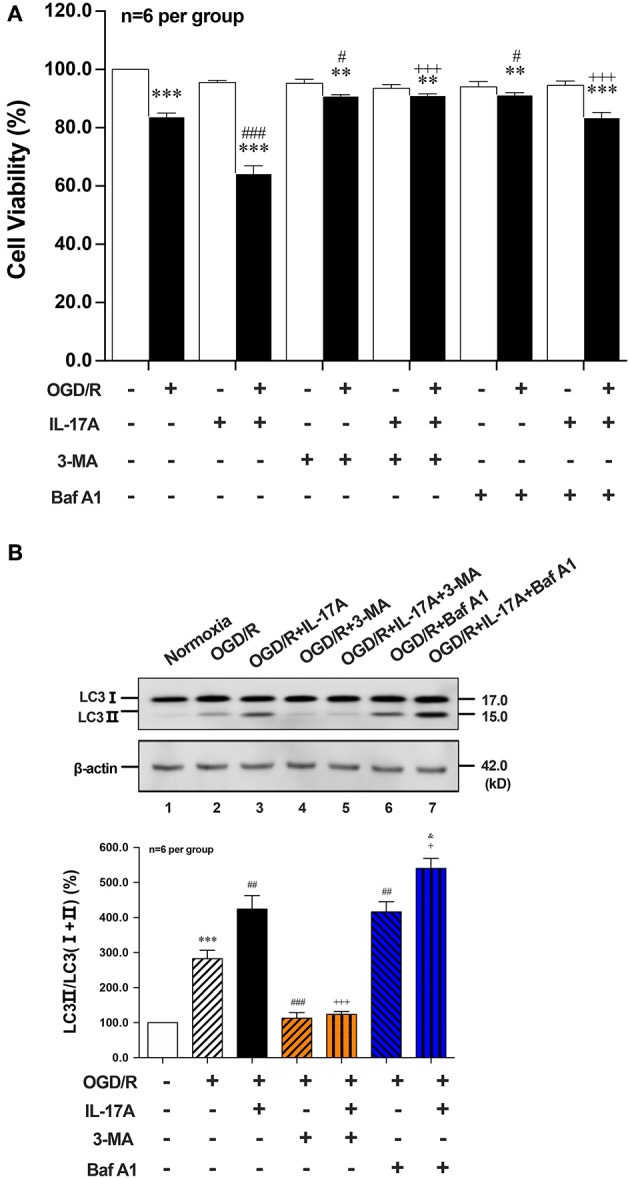
Effects of autophagic inhibitors on cell viability and the ratio of LC3 II/LC3(I+II) in OGD/R and rmIL-17A-treated cortical neurons. The autophagic inhibitors 3-MA and Baf A1 pretreatment could significantly improve the survival rates of neurons **(A)** compared with their corresponding OGD/R or OGD/R+IL-17A (*n* = 6 per group). The typical and quantitative analysis results of Western blot showed that 3-MA and Baf A1treatments could significantly decrease and increase LC3 II accumulation in neurons **(B)** exposed to OGD/R or OGD/R+IL-17A, respectively (*n* = 6 per group). **P < 0.01, ***P < 0.001 vs. corresponding Normoxia group; ^#^*P* < 0.05, ^##^*P* < 0.01, ^###^*P* < 0.001 vs. corresponding OGD/R group; ^+^*P* <0.05, ^+++^*P* < 0.001 vs. corresponding OGD/R+IL-17A group; ^&^*P* < 0.05 vs. corresponding OGD/R+Baf A1 group.

To further explore the role of IL-17A-enhanced autophagy levels in neuronal ischemic injuries, two autophagic inhibitors 3-methyladenine (3-MA) and Bafilomycin A1 (Baf A1) were applied. Both two inhibitors pretreatment could significantly improve the survival rates of neurons when compared with their corresponding OGD/R or OGD/R+IL-17A, respectively (Figure 2A). 3-MA treatment, which blocked the nucleation stage of autophagy by specifically inhibiting vacuolar protein sorting (VPS) 34 of class III phosphatidylinositol-3-kinase (PI3K) (24), could significantly decrease LC3 II accumulation in neurons exposed to OGD/R or OGD/R+IL-17A (Figure 2B). In contrast, Baf A1 could significantly increase LC3 II accumulation in neurons with OGD/R or OGD/R+IL-17A due to the blockage of the late stages of autophagy via directly inhibiting the vacuolar H^+^-ATPase (25) (Figure 2B). These results suggested that IL-17A aggravates neuronal ischemic injuries through enhancing the autophagy levels by modulating upstream targets of autophagy pathway.

### Effects of rmIL-17A on Phosphorylation Status of Positive Autophagic Pathways and Negative Regulator in OGD/R-Treated Primary Neurons

**Figure 3:**
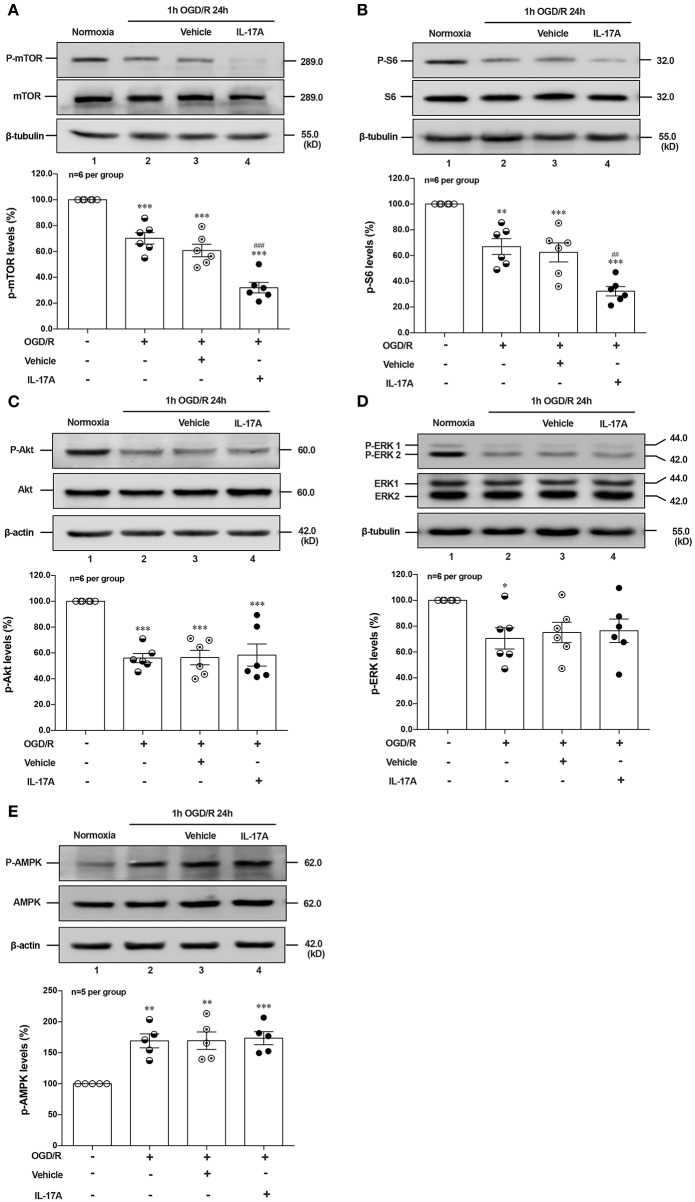
Effects of rmIL-17A on phosphorylation status of positive autophagic pathways and negative regulator in OGD/R-treated neurons. The typical and quantitative analysis results of Western blot showed that rmIL-17A could significantly promote the decrease of P-mTOR (Ser 2448, **A**) and P-S6 (Ser 240/244, **B**). However, the addition of rmIL-17A did not alter the phosphorylation status of mTOR upstream kinases P-Akt (T308, **C**), P-ERK (Y202/Y204, **D**), and P-AMPK (T172, **E**) in OGD/R-treated primary neurons (*n* = 6 per group). *P < 0.05, **P <0.01, ***P < 0.001 vs. corresponding Normoxia group; ^##^*P* < 0.01, ^###^*P* < 0.001 vs. corresponding vehicle group.

Induction of autophagy was inhibited by interaction between ULK1 and mTOR (26), but mTOR suppression could lead to the dissociation of ULK1, thus stimulating autophagy (27). To determine whether autophagy was induced upon IL-17A treatment, we evaluated the phosphorylation status of mTOR and its downstream target S6 ribosomal protein. As shown in Figures 3A,B, rmIL-17A could significantly promote the decrease of P-mTOR (Ser 2448) (A) and P-S6 (Ser 240/244) (B) levels in OGD/R-treated cortical neurons, suggesting that IL-17A causes the enhanced autophagy through mTOR dephosphorylation-mediated reduction of P-S6 (Ser 240/244) level.

To explore the underlying mechanism of IL-17A-induced mTOR dephosphorylation, the effect of rmIL-17A on mTOR upstream kinases of three classic autophagy pathways were determined in OGD/R-treated neurons, including phosphorylated (P-) Akt, P-ERK1/2 and P-AMPK. As previously shown that Akt could negatively regulate AMPK, the results showed that 1 h OGD/R 24 h treatment could obviously reduce the levels of P-Akt (Figure 3C) and P-ERK1/2 (Figure 3D), but increase the P-AMPK level (Figure 3E). However, the addition of rmIL-17A did not alter the status of P-Akt, P-ERK1/2 and P-AMPK in OGD/R-treated neurons (Figures 3C–E). Thus, neither positive regulation pathway (PI3K-Akt and ERK1/2) nor negative regulator, AMPK participated in IL-17A-induced excessive autophagy during OGD/R exposure.

### rmIL-17A Induces Excessive Autophagy via Src-PP2B-mTOR Pathway in OGD/R Treated Neurons

**Figure 4:**
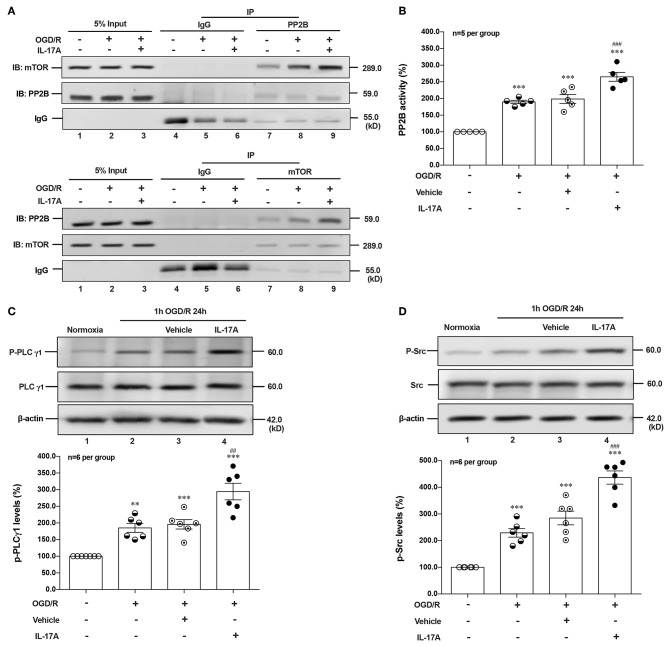
IL-17A enhances autophagy levels via Src-PP2B-mTOR pathway in neurons exposed to OGD/R. The co-immunoprecipitation (co-IP) analysis showed that PP2B and mTOR could reciprocally immunoprecipitated in neurons under normoxic or OGD/R conditions, and the addition of rmIL-17A could increase their interactions **(A)** and PP2B activities **(B)** in OGD/R-treated cortical neurons (*n* = 5 per group). In addition, the representative and quantitative analysis results demonstrated that P-PLCγ1 **(C)** and P-Src **(D)** levels were significantly increased in neurons exposed to OGD/R upon IL-17A treatment (*n* = 6 per group). **P < 0.01, ***P < 0.001 vs. corresponding Normoxia group; ^##^*P* < 0.01, ^###^*P* < 0.001 vs. corresponding vehicle group.

Given that PI3K-Akt, ERK1/2, and AMPK signal pathways didn't involve in IL-17A-induced dephosphorylation of mTOR during OGD/R exposure, suggesting certain type of serine (Ser)/threonine (Thr) phosphatase downstream of IL-17A signaling may be responsible. The Calcineurin/Protein Phosphatase (PP)2B is a Ca^2+^-associated Ser/Thr phosphatase, and has been proved physically binding to mTOR (28). In this study, the co-immunoprecipitation (co-IP) analysis exhibited that PP2B and mTOR could reciprocally immunoprecipitated in neurons under normoxic or OGD/R conditions, and the addition of rmIL-17A could increase their interactions (Figure 4A) and PP2B activities (Figure 4B) in OGD/R-treated cortical neurons, indicating the participation of PP2B in mTOR dephosphorylation upon IL-17A stimulation.

As an ubiquitous Ser/Thr phosphatase, PP2B can be activated by elevated Ca^2+^ levels and subsequent activation of calmodulin (CaM) (29). Previous studies demonstrated that phospholipase (PL) Cγ1 induced the release of Ca^2+^ from endoplasmic reticulum (ER) stores (30, 31). Given the involvement of Src protein tyrosine kinase in IL-17A signaling (32) and the interaction between PLCγ1 and Src kinase (33), we hypothesized that IL-17A-mediated T cell receptor (TCR) signaling induced Src to activate PLCγ1. In line with this, P-Src and P-PLCγ1 levels were significantly increased in neurons exposed to OGD/R upon IL-17A treatment (Figures 4C,D). These results suggested that IL-17A induces the elevated autophagy via Src-PP2B-mTOR pathway in neurons exposed to OGD/R.

### PP2B Inhibitor CsA Suppressed rmIL-17A-Induced Excessive Autophagy and Alleviates Neuronal Injuries After OGD/R

**Figure 5:**
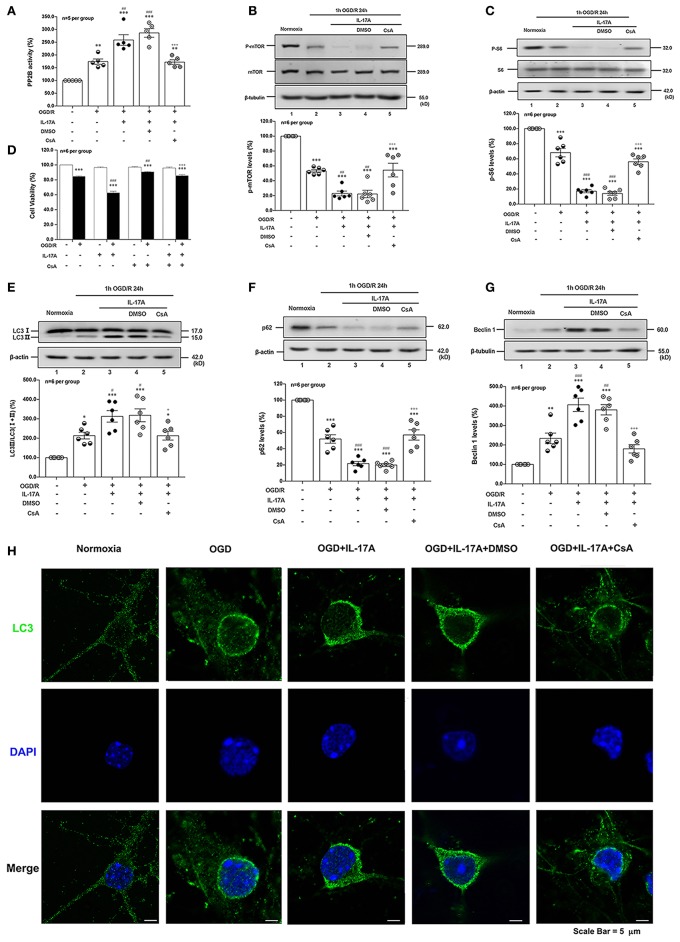
PP2B inhibitor CsA suppresses IL-17A-induced excessive autophagy and alleviates neuronal injury in primary neurons after OGD/R. The PP2B Cellular Activity Assay results confirmed that the phosphatase activities of PP2B were obviously inhibited by CsA (**A**, *n* = 5 per group). CsA could restore P-mTOR (Ser 2448) **(B)** and P-S6 (Ser 240/244) **(C)** levels, and improve neuronal cell viability **(D)** after OGD/R and rmIL-17A treatments (*n* = 6 per group). In addition, LC3 II accumulation **(E)**, p62 degeneration **(F)**, and Beclin 1 expression **(G)** levels were decreased upon CsA treatment (*n* = 6 per group). Immunofluorescent staining results showed that LC3 puncta decreased upon CsA treatment in cortical neurons **(H)** *P < 0.05, **P < 0.01, ***P <0.001 vs. corresponding Normoxia group; ^#^*P* < 0.05, ^##^*P* < 0.01, ^###^*P* < 0.001 vs. corresponding OGD/R group; ^+^*P* < 0.05, ^+++^*P* < 0.001 vs. corresponding DMSO group.

CsA, a potent immunosuppressant, inhibited the phosphatase activities of PP2B by competitive binding to cyclophilin A (34). The PP2B Cellular Activity Assay results confirmed that the phosphatase activities of PP2B were obviously inhibited by CsA (Figure 5A). The P-mTOR (Ser 2448) (Figure 5B) and P-S6 (Ser 240/244) (Figure 5C) levels were restored by CsA treatment, indicating that suppression of PP2B activity reversed the dephosphorylation of mTOR at Ser 2448. To determine the effect of PP2B inactivation on the cell survival rate, the cell viability assay was applied. As shown in Figure 5D, CsA could improve neuronal cell viability after OGD/R and rmIL-17A treatments. In addition, LC3 II accumulation (Figure 5E) and Beclin 1 expression (Figure 5G) levels were decreased upon CsA treatment, meanwhile, CsA decreased the degeneration of p62 levels (Figure 5F). Consistent with this, decreased numbers of LC3 puncta can be seen upon CsA treatment. Thus, CsA suppressed IL-17A-induced excessive autophagy via PP2B inactivation.

### Neutralization of IL-17A Reduces Autophagy Levels and Improves the Neurological Outcome of Mice With Ischemic Stroke

**Figure 6:**
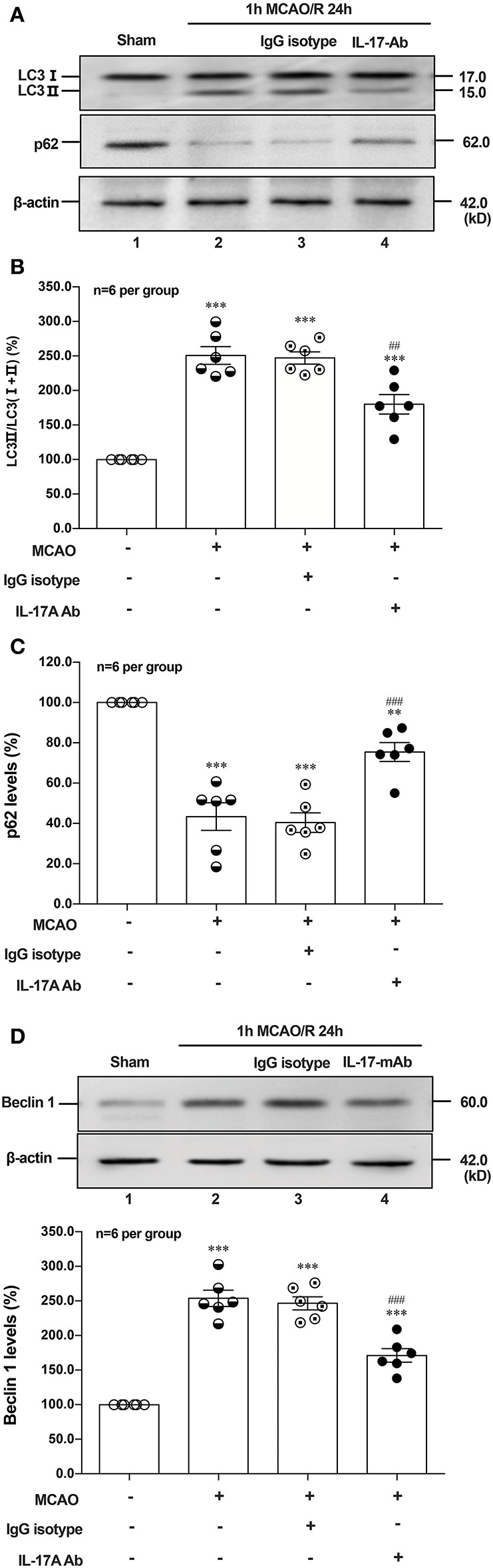
Effect of IL-17A neutralization on autophagy levels in the peri-infarct region of mice with ischemic stroke. The representative and quantitative analysis results of Western blot showed that IL-17A neutralization could significantly decrease the conversion of LC3 I to LC3 II **(A,B)** and Beclin 1 protein levels **(D)**, as well as inhibit the degradation of p62 **(C)** in the peri-infarct region of mice after 1 h MCAO/R 24 h (*n* = 6 per group). Data were presented as mean ± SEM, and One-way ANOVA followed by Bonferroni test was performed. **P < 0.01, ***P < 0.001 vs. corresponding Sham group; ^##^*P* < 0.01, ^###^*P* < 0.001 vs. corresponding IgG isotype group.

To determine the effect of IL-17A neutralizing mAb on the autophagy levels, the microtubules associated protein 1 light chain 3-β (LC3) conversion (LC3 I–LC3 II), sequestosome (p62) and Beclin 1 protein levels were detected in the peri-infarct region of mice after 1 h MCAO/R 24 h. As shown in Figures 6A,B,D, the ratio of LC3 II/total LC3 and Beclin 1 protein levels were higher than that of Sham group, but neutralization of IL-17A could significantly decrease the accumulation of LC3 II and Beclin 1 protein levels in the peri-infarct region of mice following 1 h MCAO/R 24 h. Conversely, the neutralization of IL-17A could significantly inhibit the degradation of p62 in the peri-infarct region of mice after 1 h MCAO/R 24 h (Figures 6A,C). These results indicated that the autophagy levels were downregulated by neutralization of IL-17A in the peri-infarct region of mice after 1 h MCAO/R 24 h.

**Figure 7:**
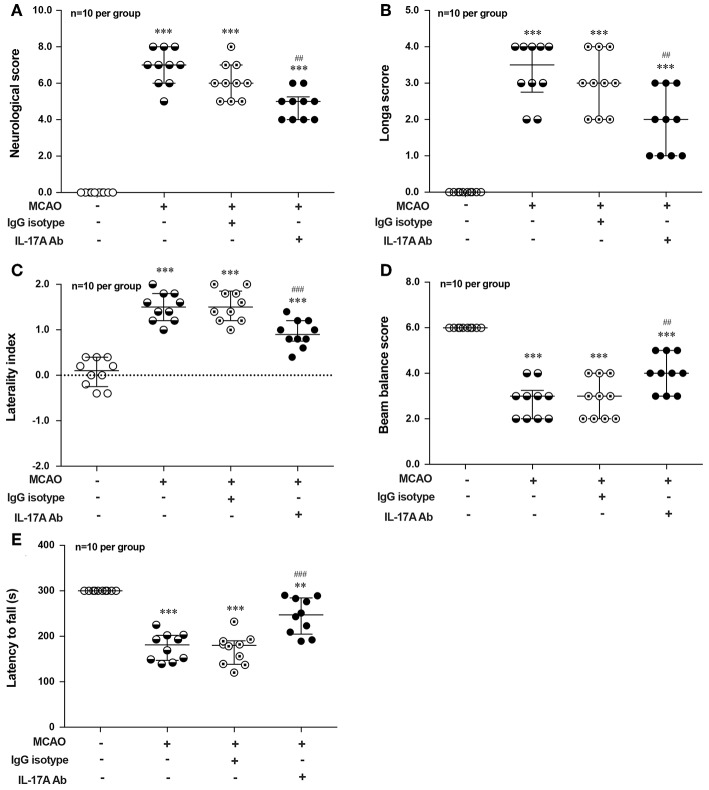
Effects of IL-17A neutralizing mAb on the neurological outcome of mice with ischemic stroke. The statistical analysis results of neurological score **(A)**, longa score **(B)**, corner test **(C)**, beam balance test **(D)**, and rotarod test **(E)** showed that neutralization of IL-17A could significantly improve the neurological functions of mice following 1 h MCAO/R 7 d when compared with that of IgG isotype group (*n* = 10 per group). Data were presented as mean ± SEM, and the statistical analysis was performed by using one-way ANOVA followed by Bonferroni test. **P < 0.01, ***P < 0.001 vs. corresponding Sham group; ^##^*P* < 0.01, ^###^*P* < 0.001 vs. corresponding IgG isotype group.

To explore the effect of IL-17A on the neurological outcome of ischemic stroke, IL-17A neutralizing mAb and mouse IgG isotype was injected into the ICV of mouse at 3 h after MCAO injury, after that neurological deficits and motor coordination were evaluated. Results of the neurological score (Figure 7A), the Longa score (Figure 7B), corner test (Figure 7C), beam balance test (Figure 7D), and rotarod test (Figure 7E) showed that the neurological function obviously decreased in mice after 1 h MCAO/R 7 d when compared with that of the corresponding Sham group. However, neutralization of IL-17A could significantly improve these neurological functions of mice following 1 h MCAO/R 7 d compared with the IgG isotype group (*n* = 10 per group).

## Discussion

Accumulating evidence suggest that IL-17A plays a particular role in the delayed phase of the post-stroke inflammatory response (4, 6, 35); the neutralization of IL-17A was proved to be a potential therapeutic measure for ischemic stroke (6). Our previous results also demonstrated that IL-17A levels in peri-infarct cortex homogenates, CSF and serum were significantly increased in mice with ischemic stroke (5); and the blockade of IL-17A with neutralizing antibody improved the neurologic outcome of mice after ischemic stroke (7). Consistent with prior observations, we further demonstrated that IL-17A-mediated excessive autophagy aggravated neuronal ischemic injuries via Src-PP2B-mTOR pathway, and IL-17A neutralization could improve the neurological outcomes of mice with ischemic stroke.

Autophagy is a double-edged sword in ischemic stroke. Basal autophagy helps cells to produce adequate energy against stressful circumstances and promotes cell survival by controlling the clearance and reuse of intracellular components. When the stress is too excessive and exceeds the maximum cellular adaptive capacity, autophagy induces cell death. Whether autophagy is beneficial or detrimental depends upon the extent of autophagy induction and the duration of autophagy activation (36). Administration of 3-MA or Baf A1 largely protected them from cell death in primary cultured cortical neurons and significantly reduced MCAO/R-induced brain infarct volume, brain edema and motor deficits, suggesting autophagy contributes to cell death both *in vitro* and *in vivo* (16, 17, 37). In line with this, our results showed that autophagy was induced in OGD/R-treated neurons. Moreover, 3-MA and Baf A1 pre-treatment ameliorated OGD/R-induced cell death. In contrast, neuronal autophagy upon ischemic injury could be a part of pro-survival signaling which is associated with the activation of PI3K-Akt-mTOR axis (38). These conflicting results are attributable to the variation in dosage and administration routes of pharmacological agents and different animal models.

In this study, the enhanced autophagy was observed in peri-infarct region of mice after 1 h MCAO/R 24 h; and according to our previous report that IL-17A was elevated in brain homogenates and CSF after 1 h MCAO/R 12 h (5), the role of IL-17A on neuronal autophagy was further explored after ischemic stroke. Several lines of evidence demonstrated that γδ T cells, T helper (Th) 17 cells as well as CNS-resident cells astrocytes and microglia, rather than neurons, are responsible for IL-17A production (39–41). Thus, OGD/R treatment with the presence of IL-17A was employed to simulate *in vivo* ischemic stroke. We found that IL-17A aggravated OGD/R-treated ischemic injuries of primary neurons by modulating the initiation of autophagic process.

**Figure 8:**
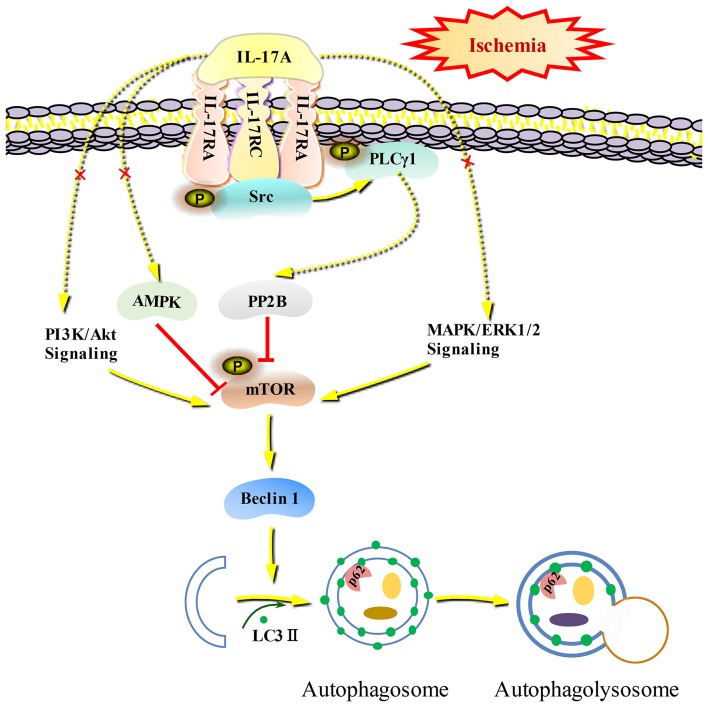
Schematic diagram of IL-17A induces excessive autophagy via Src-PP2B-mTOR pathway in neurons under ischemic condition. IL-17A stimulates IL-17A receptor-mediated signaling pathway by binding with IL-17A receptor complex IL-17RA/RC, recruiting and phosphorylating Src kinase. P-Src-mediated PLCγ1 activation could increase PP2B activity, and then the activated PP2B induces excessive autophagy through dephosphorylating mTOR.

Autophagy is a self-eating cellular catabolic pathway, which is orchestrated by a complex signaling network (8). The Ser/Thr protein kinase mTOR is a key negative regulator in autophagy initiation (42). Our results showed that the phosphorylation levels of mTOR and its latter downstream target S6 were obviously decreased in primary neurons following OGD/R. Moreover, rmIL-17A treatment caused more reduction in P-mTOR (Ser 2448) and P-S6 (Ser 240/244). Previous studies have reported that Ser 240/244 phosphorylation of S6 is regulated predominantly via an mTOR-dependent mechanism (43, 44). AKT/protein kinase B (PKB) and ERK1/2, two key upstream kinases of mTOR, can positively regulate mTOR phosphorylation and thus inhibiting autophagic flux. In addition, mTOR can be inhibited by AMPK which controls intracellular energy status by sensing the AMP/ATP ratio. We found that the addition of rmIL-17A didn't affect the P-Akt (T308), P-ERK1/2 (Y202/Y204), and P-AMPK (T172) in OGD/R-treated primary neurons, suggesting neither PI3K-Akt/ERK1/2 nor AMPK signaling pathways were involved in IL-17A-induced excessive autophagy during OGD/R treatment. Thus, we speculated that some type of Ser/Thr phosphatase participated in the process. It is well-known that PP2B plays essential roles in various processes including immune responses, nerve cell signaling and heart activity, and is the target of several therapeutic drugs that restrains the immune system (45). Previous results showed that PP2B could physically bind to mTOR via its PxIxIT motif, further proving mTOR as a direct substrate for PP2B-mediated dephosphorylation (28). Furthermore, our co-IP analysis and PP2B activity results exhibited that the addition of rmIL-17A could increase the interactions between PP2B and mTOR and PP2B activities in OGD/R-treated cortical neurons, indicating the participation of PP2B in mTOR dephosphorylation upon IL-17A stimulation. Elevation of intracellular calcium caused by ischemia and activation of CaM could remove an autoinhibitory helix from the active site of the phosphatase. PP2B plays a key role in activating Ca^2+^ signal transduction pathway. In this study, we found that IL-17A enhances autophagy level through Src-PP2B-mTOR pathway, which aggravates ischemic neuronal injury (Figure 8).

In conclusion, we firstly reported a new molecular mechanism that proinflammatory IL-17A mediated excessive autophagy to aggravate neuronal ischemic injuries via Src-PP2B-mTOR pathway. Moreover, IL-17A neutralization could improve the neurological outcomes of mice with ischemic stroke, which may provide a potential therapeutic effect for ischemic stroke in clinic.

## Data Availability Statement

The datasets generated for this study are available on request to the corresponding author.

## Ethics Statement

The animal study was reviewed and approved by the Experimental Animal Ethics Committee of the Capital Medical University (SCXK2016-0006).

## Author Contributions

TL performed the main experiments, analyzed the data and wrote the rough draft. SH, QD, JZ, and CL analyzed the data and made some of the charts. SL and JL designed the research, finalized the manuscript and received funds. All authors read and approved the final manuscript.

### Conflict of Interest

The authors declare that the research was conducted in the absence of any commercial or financial relationships that could be construed as a potential conflict of interest.
